# Protocol and establishment of a Queensland renal biopsy registry in Australia

**DOI:** 10.1186/s12882-020-01983-7

**Published:** 2020-08-01

**Authors:** Joseph Patrick Burke, Manaf Aljishi, Leo Francis, Wendy Hoy, Dakshinamurthy Divi, Roy Cherian, Jeremy Frazier, Glenda Gobe, Pedro Gois, Sridevi Govindarajulu, Sonny Huynh, Shilpanjali Jesudason, George John, Krishan Madhan, Andrew Mallett, Valli Manickam, Clyson Mutatiri, Shu-Kay Ng, Zaw Thet, Peter Trnka, Sree Krishna Venuthurupalli, Dwarakanathan Ranganathan

**Affiliations:** 1 Kidney Health Service, Royal Brisbane and Women’s Hospital, Brisbane, QLD Australia; 2 Kidney Health Service, Rockhampton Base Hospital, Rockhampton, QLD Australia; 3 Anatomical Pathology, Royal Brisbane and Women’s Hospital, Brisbane, QLD Australia; 4 School of Medicine, University of Queensland, Brisbane, QLD Australia; 5 Centre for Chronic Disease, Brisbane, QLD Australia; 6 National Health and Medical Research Council Chronic Kidney Disease Research Centre of Excellence, Brisbane, QLD Australia; 7 Kidney Health Service, Gold Coast University Hospital, Gold Coast, QLD Australia; 8 Kidney Health Service, Mackay University Hospital, Mackay, QLD Australia; 9 Kidney Health Service, Logan Hospital, Brisbane, QLD Australia; 10 Kidney Health Service, Toowoomba Base Hospital, Toowoomba, QLD Australia; 11 Kidney Health Service, Royal Adelaide Hospital, Adelaide, SA Australia; 12 Kidney Health Service, Hervey Bay Hospital, Hervey Bay, QLD Australia; 13 Kidney Health Service, Townsville Hospital, Townsville, QLD Australia; 14 Kidney Health Service, Bundaberg Base Hospital, Bundaberg, QLD Australia; 15 School of Medicine, Griffith University, Brisbane, QLD Australia; 16 Kidney Health Service, Queensland Children’s Hospital, Brisbane, QLD Australia

**Keywords:** Renal biopsy, Renal, Registry, Queensland, Australia, Kidney diseases

## Abstract

**Background:**

Renal biopsy is often required to obtain information for diagnosis, management and prognosis of kidney disease that can be broadly classified into acute kidney injury (AKI) and chronic kidney disease (CKD). The most common conditions identified on renal biopsy are glomerulonephritis and tubulo-interstitial disorders. There is a paucity of information on management strategies and therapeutic outcomes in AKI and CKD patients. A renal biopsy registry will provide information on biopsy-proven kidney disorders to improve disease understanding and tracking, healthcare planning, patient care and outcomes.

**Methods:**

A registry of patients, that includes biopsy-proven kidney disease, was established through the collaboration of nephrologists from Queensland Hospital and Health Services and pathologists from Pathology Queensland services. The registry is in keeping with directions of the Advancing Kidney Care 2026 Collaborative, established in September 2018 as a Queensland Health initiative. Phase 1 of the registry entailed retrospective acquisition of data from all adult native kidney biopsies performed in Queensland, Australia, from 2002 to 2018. Data were also linked with the existing CKD.QLD patient registry. From 2019 onwards, phase 2 of the registry involves prospective collection of all incident consenting patients referred to Queensland public hospitals and having a renal biopsy. Annual reports on patient outcomes will be generated and disseminated.

**Discussion:**

Establishment of the Queensland Renal Biopsy Registry (QRBR) aims to provide a profile of patients with biopsy-proven kidney disease that will lead to better understanding of clinico-pathological association and facilitate future research. It is expected to improve patient care and outcomes.

## Background

Kidney disease is an increasing healthcare problem both globally and in Australia. Kidney injury can be acute kidney injury (AKI) or chronic kidney disease (CKD). AKI is estimated to occur in 16.9% of hospital episodes in Australia and New Zealand [1], and subsequent renal function decline is greater after AKI. Overall, 25% of AKI patients progress to CKD, even if post-discharge kidney function returns to normal [2]. The first national report on AKI in Australia has shown that AKI is a growing problem that disproportionally affects those residing in socioeconomically disadvantaged areas who have higher AKI hospitalisation and death rates [3]. National renal biopsy registries provide useful information in biopsy-confirmed AKI. Spanish data from a renal registry study collected over a decade showed that 16.1% of the biopsies were diagnosed with AKI, with the prevalence order of biopsy-confirmed acute renal failure according to aetiology as vasculitis (23.3%), acute tubulointerstitial nephritis (11.3%) and crescentic glomerulonephritis (GN) (10.1%). This study also highlighted that the prevalence of the different causes differed significantly according to age group [2].

Approximately 1.7 million Australian adults have CKD, with 17,000 deaths with CKD listed as an underlying or associated cause of death in 2017 [4]. The most common causes of CKD in Australia are diabetic kidney disease, glomerulopathy, tubulointerstitial disorders of the kidney and hypertensive nephropathy. The incidence of GN is about 0.7–2.8 per 100,000/year [5] and is the second leading cause of end-stage kidney disease (ESKD) requiring kidney replacement therapy in Australia and New Zealand [6]. These disorders, therefore, pose a significant burden to the health care system as the risk of progression to ESKD is high. The annual cost of hospital haemodialysis per ESKD patient in Australia is estimated to be around AUD$ 80,000 [7].

There is a growing interest and research on the epidemiology of GN to shed light on its trends, environmental origins and pathogenesis, and generate hypotheses for novel treatment options. An increasing incidence of rapidly progressive crescentic GN over the last decade has been observed, with 75% of it due to anti-neutrophil cytoplasmic antibody-associated GN (ANCA GN) [8]. Gross variation in biopsy proven ANCA GN has been found across two metropolitan Queensland hospitals although they provide services to population of similar size of about one million each. Silica dust from mining industry is considered as a risk factor for ANCA GN [9, 10]*.* The exact reasons for the increasing trend or for disparity in different locations are not known. Aboriginal adults have an increased incidence of CKD and ESKD [11]. The reasons are not entirely understood, but may be explained by low birth weight and infections causing GN in childhood [12]. Lupus nephritis, another form of GN, has been shown to predominantly affect Australians of Asian descent [13]. Differences in prevalence of GN among populations could also be explained by genetic factors. For example, defects in alternative complement pathway may be involved in some forms of GN such as C3 glomerulopathy, atypical haemolytic-uraemic syndrome, ANCA-related GN and immunoglobulin A nephropathy [14]. Even though there is some information that genetics, geography, environment, race and socio-economic conditions play a role, data are inconclusive on the longitudinal course, management strategies and therapeutic outcomes of these patients [7]. There is also little clinical or research collaboration and lack of long-term studies from Australia on the outcome of biopsy-proven acute kidney disorders or tubulointerstitial nephritides. A comprehensive database or registry, including patient demographics, co-morbidities, laboratory investigations, histological diagnosis, treatment and outcome of biopsy proven renal disorders, is therefore required.

The creation of a renal biopsy registry can facilitate conducting high quality trials [15]. Kidney (or renal) biopsy registries have been established in many countries to understand kidney disease epidemiology, enhance nephrologists’ collaboration to improve patient care, and evaluate new clinical interventions [1, 16]. Several renal biopsy registries exist to address the natural history of kidney disease, describe the clinical features, evaluate treatment, understand risk factors for complications, and support studies and health services research in these disorders. Examples include the Danish Renal Biopsy Register [17], the Canadian Glomerulonephritis Registry [18], the Czech Registry of renal biopsies [19], the Spanish Registry of Glomerulonephritis [20], the Italian Registry of Renal Biopsy [21] and Japan Renal Biopsy Registry [22]. There is no established renal biopsy registry in Australia. Data on the epidemiology of biopsy proven GN was first published from Victoria in 2001 [23] and 15 years later from Queensland [8]. There have been no significant changes over time with age, gender or incidence of biopsy proven GN in Australia [8]. A recent study on biopsy proven GN from New Zealand has shown age, hypertension and heavy proteinuria at diagnosis are strong predictors of progression to ESKD and death [24]. Nevertheless, there is a paucity of data and high-quality clinical trials on GN on its pathogenesis, management strategies and therapeutic outcomes. Some reasons for this are lack of consensus in pathological definitions, low prevalence of disease, difficulty in recruitment, high costs of trials and lack of collaboration. Renal biopsy registry can facilitate such research and provide platform for patient recruitment and research collaboration.

Renal transplant histology is variably obtained at surgery after clamp release. Allograft biopsy is more frequently performed when acute or chronic rejection, recurrence of native disease, de novo GN, tubulointerstitial nephritis, calcineurin toxicity, microangiopathy and unexplained renal dysfunction are considered. Some centres perform protocol biopsies as well. A state-wide data registry can show the varying immunosuppression used and its effects toward allograft and patient health and provide valuable information in optimising therapy.

Queensland has a population of just over 5 million, a mix of socioeconomic status that includes indigenous Australians and peoples that are culturally and linguistically diverse. Queenslanders live in metropolitan cities, smaller towns and rural and remote areas. Queensland is approximately fifth of the country by size and has approximately fifth of the dialysis population. As a state, Queensland is representative of both the national Australian population in general, and the renal population. This proposed Renal Biopsy Registry is in line with the Advancing Kidney Care 2026 (AKC2026) Collaborative, which was launched in September 2018 by Queensland Health with the aims of standardising care, improving access and aligning efforts and resources for people with kidney disease within public renal services through a state-wide funding model that links funding to outcomes. The core of the AKC2026 is establishing strategies for effective prevention, timely detection, equitable access to patient-centred high-quality care and improved specialist kidney care, including a focus on Aboriginal and Torres Strait Islander and rural and remote needs. The registry will be a component of a strategy to better enable Queensland Health to collect and report information on patients and the provision of kidney services across the state, thereby reducing variation in outcomes, helping monitor kidney care and supporting equitable service access.

## Methods

### Registry aim and objectives

The principal aim of the Registry is to establish a registry of patients with biopsy-proven renal disorders to improve patient care and outcomes. The Registry achieves this by profiling renal biopsy proven disorders in Queensland.

Specific objectives include:

- Development of a prospective collection of clinical, laboratory, pathology, treatment and outcome data of patients with biopsy proven medical renal disease
- Consolidation of data into a collated data set as a registry
- Evaluation of patient data to identify and facilitate improved clinical care and management
- Identification and development of health policies targeting patients with biopsy proven renal disorders
- Support of clinical research
- Development of links and collaborations with other registries nationally and internationally

**Fig. 1:**
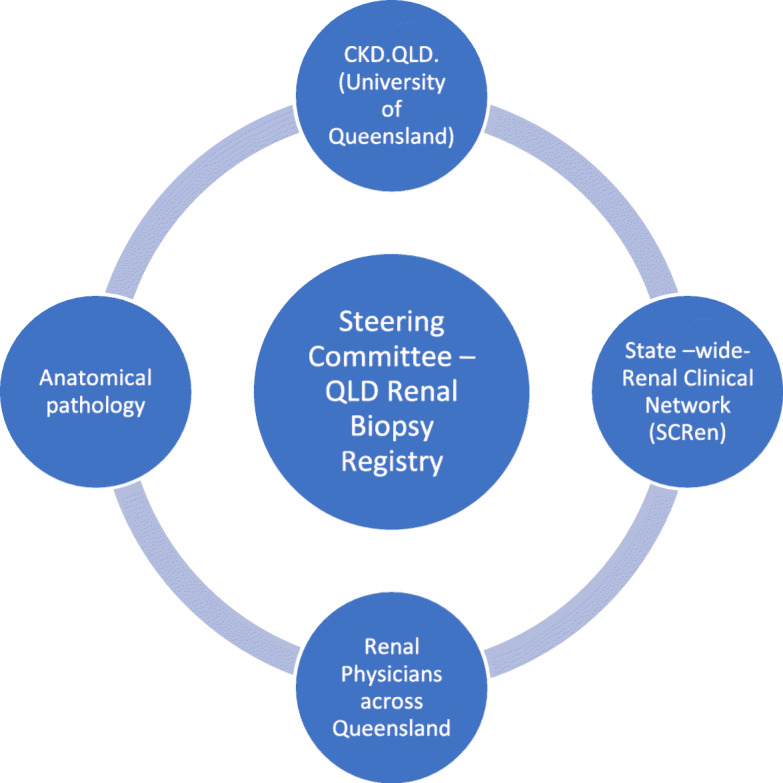
The Queensland Renal Biopsy Registry governance and collaboration network

The Queensland Renal Biopsy Registry (QRBR) is a collaborative effort between Renal Physicians from Hospital and Health Services, the State-wide Clinical Renal Network [SCReN], Pathology Queensland and CKD.QLD registry [The University of Queensland] (Fig. 1). The QRBR Steering Committee reports to SCReN through the Chair of the committee.

### Registry project

#### Phase 1: retrospective analysis of data collected for the study on epidemiology of biopsy proven glomerulonephritis in Queensland

This retrospective analysis reviewed the data from all adult native renal biopsies performed in Queensland public hospitals from 2002 to 2018--comparing results with centres from across the world. Pathology reports of 3697 adult native kidney biopsies were reviewed, of which 2048 had GN diagnoses. Age, gender, clinical indication and histopathology findings were compared [8]. Further analysis of this data will provide information on treatments administered and outcome of the patients with glomerular disorders.

#### Phase 2: collection of data prospectively

Data of all incident consenting patients with biopsy proven renal disorders referred to the above public hospitals are collected from 2018. Of note, an average of 300 kidney biopsies are performed annually in the public [government funded] sector, each of which is stored in a single Queensland Health laboratory system. The data of incident patients with diagnosed biopsy-proven renal diseases are from Queensland public health.

About 13% of the patients who consented to CKD.QLD Registry had a renal biopsy. Their data are linked to QRBR data to allow further understanding of clinical and epidemiological parameters.

### Ethics and research governance approvals

Multi-site ethics approval for the nine public hospitals was obtained from the Royal Brisbane and Women’s Hospital (RBWH) Human Research and Ethics Committee. Subsequently individual hospitals’ research governance office gave permission to conduct the study. Data collection has also been approved in accordance with the Australian Public Health Act. Further details of approvals are provided at the end of this document.

### Registry participants for prospective data collection

Patients undergo a full clinical workup for their kidney disease as part of their usual care by their treating nephrologist, who may then decide to investigate with a renal biopsy. Patients who require a renal biopsy are consented for the procedure by their nephrologist or registrar, in clinic or in a hospital setting. At the time of consenting for the risks of the renal biopsy the patient is consented to take part in data collection for the QRBR. Patients are later given the results of the biopsy by their nephrologist as they would otherwise, per routine care.

In Queensland, kidney biopsies are currently reported at 2 pathology centres, one for northern health services and one for southern services. There is discussion of biopsy results at a monthly meeting to allow consensus between nephrologists and pathologists on the diagnosis. Diagnosis is reported using the ANZDATA diagnosis coding, allowing standardisation of reporting.

### Inclusion criteria for prospective data collection

- All children or adults with renal biopsy proven medical renal disease

### Exclusion criteria for prospective data collection

- Not willing to participate in the registry
- Inability to obtain informed consent from the parent or guardian
- Inability of the patient to understand English and no interpreter available to translate

### Data access

QRBR encourages communication from anyone interested in using registry data for research purposes. All request will be considered provided they fall within the guidelines and ethical parameters of the Registry (please see Additional file 1).

### Data collection

#### Sites of data collection

Prospective data are currently being collected by the health services at RBWH, Rockhampton, Mackay and Bundaberg. Other centres are in the process of obtaining governance approval.

#### Method of data collection

At time of consenting the patient for a renal biopsy procedure, clinicians at participating centres recruit patients to the QRBR. Consent includes patient agreement to release of clinical of information. Types of clinical observation data are described below. These are collected by a researcher using a standardised data collection form (see Additional file 2), this is then entered into an online database tool (Research Electronic Data Capture, REDCap). As Queensland is moving towards electronic health records with 80% of hospitals utilising integrated electronic medical record (iEMR), in the future data will be collected via digital extraction avoiding manual entry; this will improve efficacy and accountability.

#### Collected data

No blood or tissue sample is collected or stored

- Patient demographic data including gender, age, weight at birth, ethnicity, postcode, current and past occupations.
- Co-morbidities, date of diagnosis, clinical features, pathology including serial renal function tests, immunological tests, imaging results, and renal biopsy.
- Subsequent renal management: Medications, plasmapheresis and dialysis if performed, time from referral to date of renal biopsy, time from referral to initiation of treatment.
- Subsequent referral patterns: renal function at referral to renal service and referral history.
- Subsequent patient outcomes: disease progression, commencement of renal replacement therapy.
- Subsequent death.

### Data analysis

Descriptive statistics and frequency distributions will be done for continuous and categorical variables respectively. The association between renal function and risk factors [e.g. age, sex, time taken for referral, renal function on presentation], will be calculated and analysed by bi-variate [unadjusted] and multivariate analysis [adjusted].

All analyses will be undertaken using Stata 13.1 [Stata Corp. Stata Statistical Software: Release 13.1 College Station, Texas].

Regional mapping of biopsy proven renal cases across Queensland will be done using ARCGIS [Aeronautical Reconnaissance Geographic Information System ARCIS] to identify possible regional clustering which could give hints on aetiology, including possible exposures.

### Registry timeframe

Data from renal biopsy collected between 2012 and 2018 has already been collected retrospectively and added to the registry. From 2019 onward data will be collected prospectively.

### Sample size

We estimate that 300 biopsy proven renal disorder patients are seen annually in public hospitals. For example, there will be about 600 patients in 2 years. This sample size will be generally sufficient for association tests in regression analyses with more than 20 independent variables and for estimating the population mean or proportion with a small margin of error.

### Data security and retention

Data are stored in secure locked locations on Queensland Health government property at each participating hospital. The primary central data repository is located within the secured electronic filing system of the Metro North Kidney Health Service.

Physical Security: All computers which store electronic health information data are contained in locked rooms with limited, staff ID-based, restricted access.

Technological Security: All study related documentation and participant data/data bases is stored on a secure limited-shared file. Access to shared files within Queensland Health is restricted and password protected. The computers of all persons who can access health information data are protected with automatic screen locking after 5 min of inactivity. In accordance with the high-quality security provisions of Queensland Health, computers and the electronic information that is stored upon them will be protected using firewalls, secure encrypted pathways or other methods as recommended by the Information Technology department. All security protection measures are regularly updated.

Paper records: no paper records of any personal health information data are created, at any time.

### Data reporting and dissemination

At a minimum of annual intervals during the study, results are converted into a report of de-identified data for user groups, participating Renal units, and collaborators, which will include, but is not limited to, Queensland Health, The University of Queensland, the Australasian and New Zealand Society of Nephrology, the Renal Society of Australasia, and Kidney Health Australia. In addition, outcomes will be published in academic journals and on the QRBR website: www.QRBR.org, for professional and patient information and dissemination. Reports of the units will be publicly displayed if there is a minimal collection of the data of at least 80% of the patients.

## Discussion

### Recruitment process

Recruitment has progressed since 2012, across healthcare centres in Queensland, including retrospective data from approximately 3500 renal biopsies. As of 2019, the registry has included prospective data from approximately 100 patients. The majority of data have been collected from the Royal Brisbane and Women’s Hospital (RBWH). There are plans to expand data collection practices in 2020. Recruitment is currently limited due to lack of resources in data collection and in consenting patients to enrol in the registry. Currently the consent process is undertaken by treating renal practitioners.

### Major strengths

One major strength of the registry is that it is currently the first of its kind in Australia; there are no other published databases including renal biopsy data from an Australian cohort. This registry will allow longitudinal scope of biopsy-proven renal disorders in a unique population of patients with different ethnic, socio-economic, environmental influences than existing renal biopsy registries in America, Europe and Asia [1, 18–20, 23].

Several cohort trials have been carried out on prospective data from the registry, a list is available of the QRBR website (https://qrbr.jimdosite.com/projects/).

### Limitations

Currently data collection is limited to patients in Queensland who undergo renal biopsy within the public health system. Privately collected specimens cannot be included until the registry framework is expanded, requiring further ethics approval and resources for consenting patients. Additionally, a small number of hospitals within the public health system are not yet included in the registry. There also exists an indication bias, as the data only includes patients in whom a renal biopsy was deemed indicated by their physician, therefore not capturing the disease of patients not deemed to require a biopsy or where a biopsy was contra-indicated for other reasons.

Specimens are collected from patients who underwent a renal biopsy as part of their clinical practice. Results could therefore be confounded by cases where renal biopsy was technically difficult, not deemed clinically indicated, contra-indicated due to patient factors, or when patient refuses.

Data and subsequent analyses from this registry are liable to possible indication bias.

### Future scope

There is a plan to apply for more funding for the Queensland Renal Biopsy Registry. This would allow increased recruitment of patients and to expand recruitment to other centres. In the future, further collaboration with other states could allow the registry to expand to a nationwide database. This would require significant coordination due to logistical and software differences in healthcare systems that exists between Australian states.

## Conclusions

The Queensland Renal Biopsy Registry is the first state-wide registry of renal biopsy data in Australia. Analysis of the database provides opportunities for better understanding of the longitudinal course of renal disorders. The registry provides opportunity for collating of epidemiological data on geographic, socioeconomic and ethnic factors which may contribute to renal disease. This will facilitate research and provide a single access point of data for treating clinicians, patients and their families. By providing an improved understanding of renal disease, the registry will support better patient care, management decisions, research and health economics.

## Supplementary information

**Additional file 1.**

**Additional file 2.**

## Data Availability

We encourage submission for researchers interested in obtaining data for analysis. Please contact principal investigators via email with expression of interest (see Additional file 1).

## Abbreviations

AKC2026: Advancing Kidney Care 2026
AKI: Acute kidney injury
ANCA: Anti-neutrophil cytoplasmic antibody
CKD: Chronic kidney disease
ESRD: End-stage renal disease
GN: Glomerulonephritis
QLD: Queensland
QRBR: Queensland Renal Biopsy Registry
SCReN: State-wide Clinical Renal Network
