# Supplementary material for: Protocol and establishment of a Queensland renal biopsy registry in Australia
Source: BMC Nephrol. 2020 Aug 1;21:320. doi: 10.1186/s12882-020-01983-7 (PMC7395341; doi:10.1186/s12882-020-01983-7)
Supplement: Supplementary file 1 — Additional file 1. [file 12882_2020_1983_MOESM1_ESM.docx]

QUEENSLAND RENAL BIOPSY REGISTRY: Data Access Guidelines 2019

*www.qrbr.org*

Queensland Renal Biopsy Registry

HREC Reference Number: HREC/17/QRBW/661

Australian Clinical Trials Registration Number: ACTRN12619001030167

**Data Access Guidelines**

The Queensland Renal Biopsy Registry (QRBR) use of its data for a variety of purposes, including quality assurance, audit, surveys, reports and research projects, and we welcome enquiries as to use and interpretation of the information we collect. We encourage communication with the Registry to help us to meet your request, particularly if you have not used Registry data before. Provided they fall within our guidelines and the ethical parameters of the Registry.

**Request Guidelines.** Data requests will be considered within three broad headings:

1. ***Nature of request***

The request should include the information sought, the usage proposed (including further analyses if appropriate) and an undertaking that the source of the data will be acknowledged. An indication of the level of the data requested should be given. (Extensive analysis of case-level data would normally be undertaken as part of a collaborative effort with the Registry. We may also provide advice about the methods of analysis or may suggest other sources of advice to ensure the best use is made of the data.

1. ***Nature of requesting party***

In general, requests from Queensland Health renal physicians, renal unit staff, charitable bodies, academic institutions, other registries and government departments are fulfilled without charge. Individual hospital outcomes will usually only be released to people from that institution with the approval of the head of the contributing department. Requests from industry and other bodies are considered on a case-by-case basis and may be subject to a processing fee.

1. ***Patient Confidentiality and Privacy Regulation***

The Commonwealth Privacy Act and the Queensland Health Services Act 1991 impacts on release of some forms of data. **An individuals’ identifiable data will not be released.**

**Conditions of Use of data**

Data is released to specific people for specific projects; use for other projects beyond the scope of the original application or release to other people or groups is not permitted without prior CKD.QLD Management approval. Security of the data is the responsibility of the researcher. It should be maintained in a secure computer storage facility.

**Acknowledgement**

If data from the QRBR is the primary source for a report or publication, a copy of this would ideally be sent to QRBR prior to publishing. It is a condition of use of the QRBR that the source of the data is acknowledged, along with a statement that the analysis and interpretation are those of the author, not the QBRR (see below).

**Attribution of Publications**

Where a member of a participating unit has analysed data provided by the QRBR and subsequently prepared a manuscript, then "Queensland Renal Biopsy Registry" should be acknowledged as a secondary institution in addition to the author’s Centre, Hospital or University. This applies whether the primary data analysis is performed by the author or by QRBR staff. Where the author is a QRBR office holder or staff member then the primary attribution should be "Queensland Renal Biopsy Registry".

Where QRBR data is only a minor portion of the work, then it may be more appropriate to acknowledge the source explicitly in the "Acknowledgements" section.

In both cases the disclaimer below should be included:

*“The data reported here have been supplied by the Queensland Renal Biopsy Registry. The interpretation and reporting of these data are the responsibility of the Authors and in no way should be seen an official policy or interpretation of the Queensland Renal Biopsy Registry.”*

In all cases the source and treatment of the data should be made clear in the "Methods" section. Preferably the abstract (and keywords if applicable) should also include "Queensland Renal Biopsy Registry" which would allow for searching Registry publications.

**Acknowledgement**

The ANZDATA Registry Data Access Guidelines and CKD.QLD Registry Data Access Guidelines were used in the development of this document.

**Contact Details**

Please email inquiries to [info@qrbr.og](mailto:info@qrbr.og) or phone +617 3646 2640. Visit the Queensland Renal Biopsy Registry website at [www.qrbr.org](http://www.qrbr.org) for further committee member details.
