# Supplementary material for: Protocol and establishment of a Queensland renal biopsy registry in Australia
Source: BMC Nephrol. 2020 Aug 1;21:320. doi: 10.1186/s12882-020-01983-7 (PMC7395341; doi:10.1186/s12882-020-01983-7)
Supplement: Supplementary file 2 — Additional file 2. [file 12882_2020_1983_MOESM2_ESM.pdf]

Demographics

Record ID

____
(* Automatically populated field)

The Record ID above is automatically generated and unique.

Patient: [d_last_name], [d_first_name]

DEMOGRAPHICS

PATIENT INCLUSION INTO THE QRBR

Inclusion criteria:
All children and adults indicated for a renal biopsy within an ethically and governance approved renal service, as listed, and able to provide informed consent.

A copy of the generic patient information and consent form (PICF), is available from your renal service coordinator.

Renal service where the patient consented to the study.

- ◯ Bundaberg - Wide Bay HHS
- ◯ Gold Coast - Gold Coast HHS
- ◯ Hervey Bay/Maryborough - Wide Bay HHS
- ◯ KHS (RBWH) - Metro North HHS
- ◯ Queensland Children's Hospital
- ◯ Logan - Metro South HHS
- ◯ Mackay - Mackay HHS
- ◯ Rockhampton - Central Qld HHS
- ◯ Toowoomba - Darling Downs HHS
- ◯ Townsville - Townsville HH

Date of Informed Consent

____
(YYYY-MM-DD)

Upload the patient's informed consent form
* For paper forms, please attach consent.

Date a copy of the consent form was provided to patient eg photocopy on the day of consent/date mailed

____
(YYYY-MM-DD)

Patients surname

____

Patients first name

____

Gender

- ◯ Female
- ◯ Male
- ◯ Other

Date of birth

____
(YYYY-MM-DD)

Country of Birth

- ◯ 1601 Adelie Land (France)
- ◯ 7201 Afghanistan
- ◯ 2408 Aland Islands
- ◯ 3201 Albania
- ◯ 4101 Algeria
- ◯ 3101 Andorra

Ethnicity

- ◯ 0000 Response Unidentifiable
- ◯ 0001 Not Stated
- ◯ 1101 Oceanian - Australian
- ◯ 1102 Oceanian - Australian Aboriginal
- ◯ 1103 Oceanian - Australian South Sea Islander
- ◯ 1104 Oceanian - Torres Strait Islander
- ◯ 1201 Oceanian - New Zealand Maori

Height (cm)	______________________________
Weight (kilograms) at the time of referral	______________________________
BMI	______________________________
Notes and general comments of interest to help staff improve patient experience.<br>E.g patient weight changes often.	______________________________________
Consent withdrawn date	______________________________<br>(YYYY-MM-DD)

Occasion Of Service

Patient: [d_last_name], [d_first_name]

OBSERVATIONS & PATHOLOGY AT TIME OF ASSESSMENT

Date of clinical assessment where a renal biopsy was indicated.	______________ (YYYY-MM-DD)
Name of unit/service performing renal biopsy<br>e.g RBWH Medical Imaging	______________
Blood Pressure systolic	______________
Blood pressure diastolic	______________
Weight in kilograms<br>e.g 110	______________
Height has been recorded as :	______________

PATHOLOGY RESULTS

Pathology Lab name	◯ Pathology Queensland<br>◯ Medlab<br>◯ QML<br>◯ SNP<br>◯ Other
Pathology Lab name if "Other"	______________
eGFR (mL/min/1.73m2)	______________
eGFR measuring unit (if different)	______________

Note:
Digital pathology data can be obtained from the source/company. It is advised that pathology data is entered last if time is of concern.

RENAL CHEMISTRY PANEL

Urea (mmol/L)	______________
Creatinine (umol/L)	______________
Potassium (mmol/L)	______________
Bicarbonate (mmol/L)	______________
Sodium (mmol/L)	______________
Calcium (mmol/L)	______________
Corrected Calcium (mmol/L)	______________
Phosphate (mmol/L)	______________

Total Protein (g/L)	____________
Albumin (g/L)	____________
Glucose (mmol/L)	____________
HbA1c (%)	____________
Total Cholesterol (mmol/L)	____________
Triglycerides (mmol/L)	____________

FULL BLOOD COUNT

Haemoglobin (g/L)	____________
White Cell Count (x10^9/L)	____________
Platelet Count (x10^9/L)	____________

Ferritin (ug/L)	____________
Transferrin (g/L)	____________
Vitamin B12 (pmol/L)	____________

URINE TESTS

Urine microscopy	☐ Leucocytes >10 (x10^9/L)<br>☐ Erythrocytes >10 (x10^9/L)<br>☐ Epithelials >10 (x10^9/L)<br>☐ Casts
U-Protein Creatinine Ratio (g/mol creat)	____________
U-Albumin Creatinine Ratio (g/mol creat)	____________
24 Hour Urine Protein (mg/24hr)	____________

MYELOMA SCREEN

Was a screening for Myeloma done?	◯ Yes<br>◯ No
Urine for free light chains	◯ Yes<br>◯ No
Serum for free Light Chains	◯ Yes<br>◯ No
U-Bence Jones Protein	◯ Detected<br>◯ Not Detected<br>◯ Not reported

GN SCREEN	
ANA	____________
ENA	____________
AntidsDNA	____________
AntiSM	____________
Anti La	____________
Anti Ro	____________
Anticardiolipin	____________
Lupus Anticoagulant	____________
Beta2 Glycoprotein	____________
Rheumatoid Factor	____________
Anti CCP antibodies	____________
antiGBM	____________
MPO	____________
PR3	____________
Cryoglobulins	○ Positive ○ Negative
Complements C3	____________
Complements C4	____________
Complements CH50	____________
Anti PLA2R antibodies	____________
ADAMST13	____________
Other Biochemistry / Blood Tests	____________

VIRGOLOGY SCREEN

Hepatitis B	◯ Reactive<br>◯ Non-reactive
HIV	◯ Positive<br>◯ Negative
Hepatitis C	◯ Reactive<br>◯ Non-reactive
CMV	◯ Positive<br>◯ Negative
BK Virus (Copies/mL)	______________

IMAGING SECTION

Date of Imaging	______________<br>(YYYY-MM-DD)
Renal Imaging summary	______________
Imaging report PDF	

MEDICATIONS

IMMUNOSUPPRESSIVE AGENTS

Methylprednisolone	◯ Yes ◯ No ◯ Ceased
Prednisolone	◯ Yes ◯ No ◯ Ceased
Cyclophosphamide Oral	◯ Yes ◯ No ◯ Ceased
Cyclophosphamide IV	◯ Yes ◯ No ◯ Ceased
Mycophenolate Sodium	◯ Yes ◯ No ◯ Ceased
Mycophenolate Mofetil	◯ Yes ◯ No ◯ Ceased
Azathioprine	◯ Yes ◯ No ◯ Ceased
Tacrolimus	◯ Yes ◯ No ◯ Ceased
Cyclosporine	◯ Yes ◯ No ◯ Ceased
Perindopril	◯ Yes ◯ No ◯ Ceased
Rituximab	◯ Yes ◯ No ◯ Ceased
Other Biological agents	◯ Yes ◯ No ◯ Ceased

ANTI-HYPERTENSIVES

Ramipril	◯ Yes ◯ No ◯ Ceased
Another ACEI	◯ Yes ◯ No ◯ Ceased
Irbesartan	◯ Yes ◯ No ◯ Ceased
Candesartan	◯ Yes ◯ No ◯ Ceased
Losartan	◯ Yes ◯ No ◯ Ceased
Another ARB	◯ Yes ◯ No ◯ Ceased
Amlodipine	◯ Yes ◯ No ◯ Ceased
Lercanidipine	◯ Yes ◯ No ◯ Ceased
Another CCB	◯ Yes ◯ No ◯ Ceased
Metoprolol	◯ Yes ◯ No ◯ Ceased
Bisoprolol	◯ Yes ◯ No ◯ Ceased
Another BB	◯ Yes ◯ No ◯ Ceased
Hydralazine	◯ Yes ◯ No ◯ Ceased
Prazosin	◯ Yes ◯ No ◯ Ceased
Another alpha blocker	◯ Yes ◯ No ◯ Ceased
Frusemide	◯ Yes ◯ No ◯ Ceased
Thiazide	◯ Yes ◯ No ◯ Ceased
Spironolactone	◯ Yes ◯ No ◯ Ceased
Other Diuretics<br>* Please enter one drug name per line.<br>E.g<br>Drug name 1 - status<br>Amiloride - ceased .<br>bumetanide - Yes.	______________________

DIABETIC MEDICATIONS

Glipizide	◯ Yes ◯ No ◯ Ceased
Metformin	◯ Yes ◯ No ◯ Ceased
Gliclazide	◯ Yes ◯ No ◯ Ceased
Insulin	◯ Yes ◯ No ◯ Ceased
Other antidiabetic drugs<br>Please enter one drug name per line.<br>E.g<br>Drug name 1 - status<br>Pioglitazone - ceased.<br>Sitagliptin - No.	______________________

TREATMENT PRESCRIBED at assessment

CRRT Continuous replacement therapy	◯ Yes ◯ No ◯ Ceased
HD Haemodialysis	◯ Yes ◯ No ◯ Ceased
PD Peritoneal Dialysis	◯ Yes ◯ No ◯ Ceased
Plasma Exchange	◯ Yes ◯ No ◯ Ceased

Renal Biopsy

**Patient: [d_last_name], [d_first_name]**<br><br>**RENAL BIOPSY**	
Date of Biopsy	____________________<br>(YYYY-MM-DD)
Name of unit/service performing renal biopsy<br>e.g RBWH Medical Imaging	____________________
Primary Nephrologist at Time of Biopsy	____________________
Age at this Biopsy	____________________
Auslab pathology number	____________________
Biopsy Type	◯ Native<br>◯ Transplant
Biopsy Procedure Type	◯ Percutaneous<br>◯ Transvenous<br>◯ Open
Clinical Indication for biopsy	◯ Nephritic Syndrome<br>◯ Nephrotic Syndrome<br>◯ Acute Kidney injury -unknown cause<br>◯ Interstitial nephritis<br>◯ CKD-unknown cause<br>◯ Acute Rejection<br>◯ CNI toxicity<br>◯ BK Virus nephropathy<br>◯ Chronic allograft Nephropathy<br>◯ Other indication - add in free text below please
Other indication (free text)	____________________
Blood Pressure Systolic pre-biopsy	____________________
Blood Pressure Diastolic pre-biopsy	____________________
Weight at Biopsy Date	____________________
BMI at Biopsy	____________________
Serum Creatinine at Biopsy	____________________
eGFR at Biopsy	____________________
Imaging Guidance	◯ Ultrasound Scan ◯ CT Scan
Biopsy needle Size	◯ 14 ◯ 16 ◯ 18
Needle Passes	____________________

Complications	☐ Haematuria<br>☐ Haematoma not requiring intervention<br>☐ AV Aneurysm<br>☐ Drop in Hb by >10<br>☐ Blood transfusion<br>☐ Bleeding needing embolization<br>☐ Bleeding needing surgery<br>☐ Kidney infection<br>☐ Clot obstruction<br>☐ Nephrectomy<br>☐ Death
Biopsy Report (PDF)	
Biopsy Electron Microscope image if available	
Renal Diagnosis	__________________________
ANZDATA coding<br>* If SLE, please add any additional classification (as per ISN/RPS) below<br>* If "Other"(code 0), please add descriptor below	○ 000 Other (Specify)<br>○ 001 Uncertain Diagnosis<br>○ 002 Lead Nephropathy<br>○ 003 Cadmium Toxicity<br>○ 004 Renal Tuberculosis<br>○ 005 Amyloid Disease<br>○ 006 Haemolytic Uraemic Syndrome<br>○ 007 Cortical Necrosis<br>○ 008 Interstitial Nephritis<br>○ 009 Congenital Renal Hypoplasia and Dysplasia<br>○ 010 Loss of Single Kidney (Trauma Surgery)
If renal diagnosis is SLE, then please indicate ISN/RPS classification	○ I<br>○ II<br>○ III<br>○ IV<br>○ V<br>○ VI
Primary Diagnosis ANZDATA Other (code 0) descriptor	__________________________

CHANGES TO MANAGEMENT POST RENAL BIOPSY*Please only record changes where applicable.

MEDICATIONS
IMMUNOSUPPRESSIVE AGENTS

Methylprednisolone	○ Yes ○ No ○ Ceased
Prednisolone	○ Yes ○ No ○ Ceased
Cyclophosphamide Oral	○ Yes ○ No ○ Ceased
Cyclophosphamide IV	○ Yes ○ No ○ Ceased
Mycophenolate Sodium	○ Yes ○ No ○ Ceased
Mycophenolate Mofetil	○ Yes ○ No ○ Ceased
Azathioprine	○ Yes ○ No ○ Ceased
Tacrolimus	○ Yes ○ No ○ Ceased
Cyclosporine	○ Yes ○ No ○ Ceased
Rituximab	○ Yes ○ No ○ Ceased
Other Biological agents	○ Yes ○ No ○ Ceased

ANTI-HYPERTENSIVES	
Perindopril	◯ Yes ◯ No ◯ Ceased
Ramipril	◯ Yes ◯ No ◯ Ceased
Another ACEI	◯ Yes ◯ No ◯ Ceased
Irbesartan	◯ Yes ◯ No ◯ Ceased
Candesartan	◯ Yes ◯ No ◯ Ceased
Losartan	◯ Yes ◯ No ◯ Ceased
Another ARB	◯ Yes ◯ No ◯ Ceased
Amlodipine	◯ Yes ◯ No ◯ Ceased
Lercanidipine	◯ Yes ◯ No ◯ Ceased
Another CCB	◯ Yes ◯ No ◯ Ceased
Metoprolol	◯ Yes ◯ No ◯ Ceased
Bisoprolol	◯ Yes ◯ No ◯ Ceased
Another BB	◯ Yes ◯ No ◯ Ceased
Hydralazine	◯ Yes ◯ No ◯ Ceased
Prazosin	◯ Yes ◯ No ◯ Ceased
Another alpha blocker	◯ Yes ◯ No ◯ Ceased
Frusemide	◯ Yes ◯ No ◯ Ceased
Thiazide	◯ Yes ◯ No ◯ Ceased
Spironolactone	◯ Yes ◯ No ◯ Ceased
Other Diuretics<br>* Please enter one drug name per line.<br>E.g<br>Drug name 1 - status.<br>Amiloride - ceased .<br>bumetanide - Yes.	________________________

DIABETIC MEDICATIONS	
Glipizide	◯ Yes ◯ No ◯ Ceased
Metformin	◯ Yes ◯ No ◯ Ceased
Gliclazide	◯ Yes ◯ No ◯ Ceased
Insulin	◯ Yes ◯ No ◯ Ceased
Other antidiabetic drugs<br>Please enter one drug name per line.<br>E.g<br>Drug name 1 - status.<br>Pioglitazone - ceased.<br>Sitagliptin - No.	________________________

TREATMENT PRESCRIBED at assessment	
CRRT Continuous replacement therapy	◯ Yes ◯ No ◯ Ceased
HD Haemodialysis	◯ Yes ◯ No ◯ Ceased
PD Peritoneal Dialysis	◯ Yes ◯ No ◯ Ceased
Plasma Exchange	◯ Yes ◯ No ◯ Ceased

Comorbidities Charlson Index And Others

Patient: [d_last_name], [d_first_name]

CO-MORBIDITIES CHARLSON INDEX

Date of assessment	______________________
Comorbidity (Choose all that are present)	☐ Myocardial infarct (+1)<br>☐ Congestive heart failure (+1)<br>☐ Peripheral vascular disease (+1)<br>☐ Cerebrovascular disease (except hemiplegia) (+1)<br>☐ Dementia (+1)<br>☐ Chronic pulmonary disease (+1)<br>☐ Connective tissue disease (+1)<br>☐ Ulcer disease (+1)<br>☐ Mild liver disease (+1)<br>☐ Diabetes (without complications) (+1)<br>☐ Diabetes with end organ damage (+2)<br>☐ Hemiplegia (+2)<br>☐ Moderate or severe renal disease (+2)<br>☐ Solid tumor (non metastatic) (+2)<br>☐ Leukemia (+2)<br>☐ Lymphoma, Multiple myeloma (+2)<br>☐ Moderate or severe liver disease (+3)<br>☐ Metastatic solid tumor (+6)<br>☐ AIDS (+6)
Other Co-Morbidities<br><br>E.g Hypertension, Dyslipidaemia, SLE, Rheumatoid Arthritis	______________________
Recent Clinic Letter	
Smoker status	◯ Current ◯ Former<br>◯ Never
Age Group<br><br>Recorded age is: [d_age]	◯ 50 - 59 (+1)<br>◯ 60 - 69 (+2)<br>◯ 70 - 79 (+3)<br>◯ 80 - 89 (+4)<br>◯ 90 - 99 (+5)
Total points:	______________________

Outcomes Rrt And Death

Patient: [d_last_name], [d_first_name]

OUTCOMES RRT & DEATH

Definitions to support outcome status depending on Diagnosis

Acute Kidney Injury
Complete recovery: Return of pre-AKI renal function, > eGFR > 60 ml/mt.

Partial recovery: NO CRRT but eGFR < 60 ml. mt >90 days.

No recovery: Persistent requirement for RRT.

Glomerulonephritis
Complete remission: is defined as a decrease in urinary protein measured over 24 hours to less than 500 mg /24 h, uPCR less than 0.5 mg/mg (50 mmol/mg normal serum albumin and stabilisation (± 25%) or improvement in serum creatinine levels at week 24 from the initial sample.

Partial remission is defined as stabilisation (± 25%) or improved renal function (but still not to normal) with reduction of proteinuria by more than 50% ranging between 300 to 3000mg /24h and a serum albumin of more than 30g/L.

Refractory to treatment: No Change in protienuria or eGFR.

Renal relapse is defined as "recrudescence of renal disease after an initial response demonstrated by a recent increase in serum creatinine by >50% with active urinary sediment and or increase in proteinuria to 3500 mg/day or greater.

Renal Transplantation
(*if biopsy diagnosis is Acute Rejection, CNI toxicity, BKVN or Chronic Allograft Nephropathy)
Resolved:

Not resolved:

CKD other than GN
Partial recovery: NO CRRT but eGFR < 60 ml.

No recovery: Persistent requirement for RRT.

Date of Outcome Assessment	______________________
Diagnosis	◯ Glomerulonephritis<br>◯ Acute Kidney Injury<br>◯ Renal Transplantation<br>◯ CKD other than GN
Outcome relating to diagnosis<br>*Please refer to the definitions above	◯ Complete<br>◯ Partial<br>◯ Relapsed<br>◯ No Recovery<br>◯ Refractory<br>◯ Resolved<br>◯ Not Resolved

Type of first RRT

	CRRT	Haemodialysis	Peritoneal Dialysis	Renal Transplantation
RRT	☐	☐	☐	☐

Date of commencement of renal replacement therapy

(YYYY-MM-DD)

Recovery of renal function (no longer requiring renal replacement therapy)

◯ Yes
◯ No

Date RRT ceased

(YYYY-MM-DD)

Date of RECOMMENCEMENT of an RRT (second RRT prescription)

(YYYY-MM-DD)

Type of Renal Transplant

◯ LUD ◯ LRD ◯ DCD
◯ DBD ◯ Tumourectomised

Transplant comments

Type of second RRT

	CRRT	Haemodialysis	Peritoneal Dialysis	Renal Transplantation
RRT	☐	☐	☐	☐

Date second RRT ceased

(YYYY-MM-DD)

DEATH INFORMATION

Date of Death

(YYYY-MM-DD)

Cause of Death if Known

Death certificate if available-pdf
